# Procedural Efficiency of Direct Stenting Versus Predilatation in Type A and B1 Coronary Lesions

**DOI:** 10.3390/jcm15208144

**Published:** 2026-10-20

**Authors:** Gazi Capar, Ahmet Guler

**Affiliations:** 1 Department of Cardiology, Istanbul Esenyurt State Hospital, Istanbul 34517, Türkiye; 2 Department of Cardiology, Istanbul Basaksehir Cam and Sakura City Hospital, Istanbul 34480, Türkiye; ahmetguler01@yahoo.com.tr

**Keywords:** Percutaneous coronary intervention, direct stenting, predilatation, drug-eluting stent

## Abstract

**Background/Objectives:** The optimal stent deployment strategy for low-complexity Type A and B1 coronary lesions remains poorly defined. We compared direct stenting (DS) and predilatation followed by stenting (PS) in elective percutaneous coronary intervention (PCI) for these lesions. **Methods:** This single-center, retrospective study included adults undergoing elective PCI with drug-eluting stents for ACC/AHA Type A or B1 lesions between June 2020 and December 2025. Procedure duration, cumulative contrast volume, and cumulative reference air kerma were compared using generalized estimating equations accounting for within-patient clustering, adjusted for clinical, lesion-related, procedural, and operator-related factors. **Results:** In total, 324 patients contributed 334 PCI observations (PS: 239; DS: 95). After adjustment, DS was associated with lower contrast volume (−18.12 mL; 95% CI −28.08 to −8.17; *p* < 0.001) and lower cumulative reference air kerma (−223.54 mGy; 95% CI −259.98 to −187.11; *p* < 0.001), whereas procedure duration did not differ significantly (−0.92 min; 95% CI −4.56 to 2.71; *p* = 0.619). Findings were consistent when restricted to the first PCI per patient. **Conclusions:** In these lesions, DS was associated with lower contrast volume and cumulative reference air kerma, without a significant adjusted difference in procedure duration. As nonrandomized observations, these findings do not establish equivalence in clinical safety or effectiveness, nor do they demonstrate cost-effectiveness.

## 1. Introduction

Percutaneous coronary intervention (PCI) with drug-eluting stent (DES) implantation is an established revascularization strategy in patients with chronic coronary syndromes [1]. Continuous refinements in stent design, delivery systems, and procedural technique have reduced adverse events and broadened the range of lesions suitable for PCI [2,3]. Consequently, attention has increasingly shifted toward optimizing procedural efficiency in routine cases [3]. In this context, the method of stent deployment remains a practical and frequently encountered decision: the stent can be implanted either after balloon predilatation of the target lesion (predilatation followed by stenting, PS) or without prior balloon dilatation (direct stenting, DS) [4].

Predilatation has traditionally been regarded as the standard approach. By modifying the plaque before stent delivery, it facilitates stent passage, allows the operator to assess lesion compliance and vessel size, and may promote more uniform stent expansion [5,6]. However, predilatation adds balloon exchanges, angiographic acquisitions, and fluoroscopy to the procedure [5,6]. Balloon injury outside the eventual stented segment (geographical miss) may also cause edge dissection and predispose to restenosis [7]. Direct stenting omits this preparatory step, thereby reducing the number of procedural steps [8]. It may therefore lower resource utilization, contrast volume, and radiation exposure [8]. In appropriately selected lesions, it may also limit balloon-induced dissection and distal embolization, because plaque material is compressed directly behind the stent struts [9,10].

These potential advantages are clinically relevant. Iodinated contrast is an established risk factor for contrast-associated acute kidney injury, particularly in patients with diabetes or reduced renal function, and minimizing contrast volume is a recognized goal during PCI [11]. Similarly, limiting patient radiation exposure is a central principle of catheterization-laboratory practice, and procedural strategies that reduce cumulative reference air kerma may contribute to this aim [12].

The choice between direct stenting and lesion preparation is influenced by lesion morphology. Severe calcification, marked vessel tortuosity and other complex anatomical features may impair device deliverability and complicate stent deployment, thereby increasing the need for appropriate lesion preparation before stent implantation [6,13,14]. Device characteristics are also relevant. Contemporary thin-strut DES platforms with lower crossing profiles and greater flexibility have improved stent deliverability and made DS increasingly feasible in suitable anatomy [15].

Earlier randomized trials and observational studies showed that DS could be performed with high procedural success and lower resource utilization, without an apparent increase in adverse events [16,17]. Subsequent studies in the DES era further supported the feasibility of this approach. However, available evidence has generally included heterogeneous clinical settings and lesion characteristics, while contemporary data specifically evaluating procedural efficiency in elective, low-complexity Type A and B1 lesions remain limited [18]. In particular, we considered that a focused comparison of DS and predilatation with respect to procedure duration, contrast utilization, and radiation exposure would provide additional insight into their procedural characteristics in this selected contemporary population.

Type A and B1 lesions, as defined by the ACC/AHA classification and its subsequent modification [19], represent low-complexity anatomy that is frequently encountered in routine elective PCI. This is the lesion subset in which DS is most likely to be feasible. Nevertheless, contemporary data comparing the procedural efficiency of DS and PS specifically in Type A and B1 lesions are limited, and the optimal deployment strategy in this lesion subset has not been clearly established [16,18]. As a result, the choice is often guided by operator preference rather than by evidence. The aim of this study was therefore to compare procedure duration, cumulative contrast volume, and cumulative reference air kerma between DS and PS in patients undergoing elective PCI with contemporary DES for ACC/AHA Type A and B1 coronary lesions.

## 2. Materials and Methods

This single-center, retrospective, observational, comparative study included patients aged ≥ 18 years who underwent elective percutaneous coronary intervention (PCI) for coronary artery disease between June 2020 and December 2025 and received drug-eluting stent implantation for Type A or B1 coronary lesions. The patient inclusion period refers to the dates of clinical procedures, whereas research-specific data extraction was performed retrospectively in December 2025, after ethics committee approval. PCI observations were classified according to the procedural strategy used as predilatation followed by stenting (PS) or direct stenting (DS).

Lesions were classified according to the ACC/AHA coronary lesion morphology criteria and the subsequent modification distinguishing B1 from B2 lesions [19]. Type A lesions were defined as discrete (<10 mm), concentric, readily accessible lesions located in a non-angulated segment (<45°), with smooth contour, little or no calcification, less than total occlusion, non-ostial location, no major side-branch involvement, and no thrombus. Type B characteristics included tubular length (10–20 mm), eccentricity, moderate proximal tortuosity, moderate angulation (45–90°), irregular contour, moderate-to-heavy calcification, total occlusion of <3 months’ duration, ostial location, bifurcation requiring double guidewires, and presence of thrombus. Type B1 lesions were defined by the presence of a single Type B characteristic [19]. Lesions with two or more Type B characteristics (Type B2) and Type C lesions were excluded. Lesion type was retrospectively classified by two investigators through review of the stored angiograms and procedural reports of the corresponding PCI episode. The investigators were blinded to the procedural strategy and clinical outcomes during lesion classification. Independent blinded core-laboratory adjudication was not performed. The study included 324 unique patients who contributed 334 PCI observations for 334 treated coronary lesions. Repeated PCI observations were present in nine patients: eight patients underwent two PCI procedures and one patient underwent three PCI procedures at separate time points during the study period, generating 10 additional PCI observations. These repeat procedures involved different coronary lesions and were considered separate procedural episodes. No patient contributed PCI observations to both treatment groups. At the unique-patient level, 235 patients were included in the PS group and 89 in the DS group; at the PCI-observation level, the corresponding numbers were 239 and 95, respectively.

The choice between direct stenting and predilatation was made by the treating interventional cardiologist during routine clinical practice according to angiographic lesion characteristics, procedural feasibility, and operator judgment. Because treatment strategy was not randomly assigned, the possibility of confounding by indication was considered in the statistical analysis and interpretation of the findings. Direct stenting was defined as stent advancement and implantation without preceding balloon dilatation, whereas the predilatation strategy was defined as balloon dilatation before stent implantation. Postdilatation was performed at the operator’s discretion and did not affect group assignment. Procedural records were reviewed for unsuccessful direct-stenting attempts, stent-delivery failure, bail-out predilatation, and crossover between strategies. No failed direct-stenting attempt requiring subsequent predilatation and no crossover from direct stenting to predilatation were identified in the study cohort. Accordingly, the initially selected and final procedural strategies were concordant.

Patients presenting with acute coronary syndrome, those treated with bare-metal stents, those with an estimated glomerular filtration rate < 30 mL/min/1.73 m^2^, and those undergoing PCI for Type B2 or Type C complex lesions were excluded. All procedures were performed by six interventional cardiologists with at least five years of interventional experience. Demographic, clinical, laboratory, angiographic, and procedural data were retrospectively obtained from the hospital’s electronic medical records and procedural records.

Procedure duration, cumulative contrast volume, and cumulative reference air kerma were considered the three principal procedural outcomes. Because of the retrospective design, no single confirmatory primary endpoint was prospectively prespecified, and no a priori sample-size or power calculation was performed; all eligible PCI observations during the study period were included. Procedure duration was defined as the interval from sheath insertion to final angiographic imaging and was recorded in minutes. Cumulative contrast volume was recorded in milliliters. Cumulative reference air kerma was automatically reported by the angiography system and recorded in milligrays (mGy). Lesion length was determined by visual angiographic estimation.

### Statistical Analysis

Demographic, clinical, and laboratory characteristics were summarized per PCI observation, because clinical and laboratory variables were recorded at the time of each procedure. Lesion characteristics and procedural variables were likewise summarized per PCI observation. Continuous variables were summarized as mean ± standard deviation or median (interquartile range), as appropriate, and categorical variables as frequencies and percentages. The distributions of continuous variables were evaluated using histograms, Q–Q plots, descriptive distributional characteristics, and the Shapiro–Wilk test. Unadjusted between-group comparisons were performed using Student’s *t*-test or the Mann–Whitney U test for continuous variables and the chi-square test or Fisher’s exact test for categorical variables.

The principal analysis included all 334 PCI observations from 324 unique patients. Generalized estimating equations with an identity link, an exchangeable working correlation structure, and robust standard errors were used, with patient identifier as the clustering variable, to account for within-patient correlation. Models were adjusted for age, sex, diabetes mellitus, estimated glomerular filtration rate, lesion type, lesion length, treated vessel, vascular access, and operator. Treated vessel and operator were entered as categorical covariates; operator was modeled as a fixed effect rather than as an additional clustering level. Adjusted between-group differences were reported with 95% confidence intervals. Multiplicity across the three principal procedural outcomes was addressed using the Holm procedure. No procedural outcome data were missing; missing laboratory values were handled using available-case analysis.

For the sensitivity analysis, second and third PCI observations were excluded, retaining only the first recorded PCI observation from each of the 324 unique patients. Multivariable linear regression with heteroscedasticity-robust standard errors was performed using the same adjustment covariates. Statistical analyses were performed using R software (version 4.1.3; R Foundation for Statistical Computing, Vienna, Austria). A two-sided *p* value < 0.05 was considered statistically significant.

The study protocol, covering the entire study period, was approved by the local ethics committee (KAEK/19.02.2025.74), and the study was conducted in accordance with the principles of the Declaration of Helsinki. For all procedures, including those performed after ethics approval, treatment decisions were made as part of routine clinical practice, and data were obtained retrospectively from electronic medical and procedural records; study participation did not influence clinical management.

## 3. Results

A total of 324 unique patients contributed 334 PCI observations for 334 treated coronary lesions. At the unique-patient level, 235 patients were included in the predilatation group and 89 in the direct-stenting group, whereas at the PCI-observation level, 239 and 95 observations were included in the respective groups. Nine patients contributed repeated PCI observations for different coronary lesions at separate time points, generating 10 additional PCI observations, and no patient contributed observations to both treatment groups. At the unique-patient level, the mean age was 60.2 ± 9.4 years, and 218 of 324 patients (67.3%) were men.

There were no statistically significant differences between the PS and DS groups in terms of age (median 59 vs. 61 years, *p* = 0.070), sex distribution, body mass index, blood pressure, or heart rate. The prevalence of major comorbidities, including hypertension (68.2% vs. 69.5%), diabetes mellitus (30.1% vs. 27.4%), hyperlipidemia (74.5% vs. 71.6%), atrial fibrillation, heart failure, and chronic obstructive pulmonary disease, was comparable between the two groups. Furthermore, no significant differences were observed between the groups with respect to laboratory parameters, including hematological indices (hemoglobin, leukocyte, platelet), renal function markers (creatinine, eGFR), lipid profile (LDL, HDL, triglycerides), or left ventricular ejection fraction (Table 1). These comparisons describe measured clinical and laboratory characteristics at the PCI-observation level and should not be interpreted as evidence that the groups were free from residual confounding.

When procedural characteristics were evaluated, no statistically significant differences were observed in access site (radial/femoral), operator distribution, or target lesion type (A/B1). The most frequently treated vessel was the left anterior descending artery (LAD) (PS: 41.0%, DS: 41.1%), with no significant difference in treated vessel between the groups (*p* = 0.221). Additionally, the use of supportive guidewires/catheters, median lesion length (12 mm in both groups), implanted stent length (PS: 19 mm, DS: 18 mm), and stent diameter (3.00 mm in both groups) were comparable. Postdilatation was numerically more frequent in the DS group (41.1% vs. 30.5%), although this difference did not reach statistical significance (*p* = 0.066), and repeat postdilatation rates were similar between groups. No failed direct-stenting attempts requiring bail-out predilatation and no stent-delivery failures resulting in crossover from DS to PS were identified.

In unadjusted comparisons of the three principal procedural outcomes, the DS group had a lower median procedure duration (27 vs. 33 min, *p* = 0.032), lower median cumulative contrast volume (125 vs. 150 mL, *p* = 0.009), and lower median cumulative reference air kerma (420 vs. 625 mGy, *p* < 0.001) than the PS group (Table 2). Mean procedure duration, however, was similar between groups (DS: 34.9 ± 17.5 vs. PS: 35.7 ± 12.9 min).

After multivariable adjustment for age, sex, diabetes mellitus, estimated glomerular filtration rate, lesion type, lesion length, treated vessel, vascular access, and operator, while accounting for repeated PCI observations within patients, direct stenting was associated with lower cumulative contrast volume (adjusted difference −18.12 mL; 95% CI −28.08 to −8.17; *p* < 0.001) and lower cumulative reference air kerma (adjusted difference −223.54 mGy; 95% CI −259.98 to −187.11; *p* < 0.001). The difference in procedure duration was not statistically significant (adjusted difference −0.92 min; 95% CI −4.56 to 2.71; *p* = 0.619). These findings remained unchanged after Holm correction, with adjusted *p* values of <0.001, <0.001, and 0.619, respectively (Table 3).

A sensitivity analysis restricted to the first recorded PCI observation per unique patient yielded consistent findings. Direct stenting remained associated with lower cumulative contrast volume (adjusted difference −16.05 mL; 95% CI −25.99 to −6.10; *p* = 0.002) and lower cumulative reference air kerma (adjusted difference −220.42 mGy; 95% CI −256.46 to −184.37; *p* < 0.001), whereas the difference in procedure duration remained nonsignificant (adjusted difference −1.00 min; 95% CI −4.44 to 2.44; *p* = 0.569) (Table 3).

## 4. Discussion

In this study, direct stenting (DS) and predilatation followed by stenting (PS) were compared in patients undergoing elective PCI for Type A and B1 coronary lesions, primarily in terms of procedural outcomes. DS was associated with lower cumulative contrast volume and lower cumulative reference air kerma. These associations remained significant after multivariable adjustment for measured clinical, lesion-related, procedural, and operator-related factors, and they were consistent in the sensitivity analysis restricted to one PCI observation per patient. In contrast, although median procedure duration was shorter in the DS group in unadjusted comparisons, this difference was not confirmed in the adjusted analyses.

Although measured clinical, angiographic, and procedural characteristics were broadly comparable between the groups, the retrospective and nonrandomized design does not exclude selection bias or confounding by indication. The decision to use DS or PS was based on operator judgment and lesion suitability. Lesions considered more suitable for immediate stent delivery may therefore have been preferentially selected for DS. Consequently, the observed differences should be interpreted as associations with procedural strategy rather than as causal effects attributable solely to direct stenting.

Traditionally, the PS strategy has been employed for mechanical lesion preparation. However, balloon dilatation may disrupt endothelial integrity through barotrauma, potentially increasing the risk of neointimal hyperplasia [10,20]. Balloon injury outside the eventual stented segment (geographical miss) may also cause edge dissection and predispose to restenosis [7]. DS avoids a separate balloon inflation and may limit microembolization by trapping plaque material behind the stent struts [10,21]. Because DS omits the predilatation step, the lower contrast volume and cumulative reference air kerma observed in the present study may plausibly reflect a reduction in the number of procedural steps required before stent implantation. These findings are directionally consistent with earlier studies reporting lower resource utilization with DS [22,23].

The absence of a significant difference in procedure duration after adjustment deserves comment. One possible explanation is that any time saved by omitting predilatation may represent only a small proportion of the total procedure duration. Because the recorded procedure time encompassed the entire PCI procedure rather than the stent-deployment step alone, differences attributable specifically to the deployment strategy may have been diluted. In addition, the skewed distribution of procedure duration may have further limited the ability to detect modest between-group differences. The difference in medians was not accompanied by a difference in means, suggesting that the unadjusted finding was driven by distributional characteristics rather than a consistent shift across procedures. Accordingly, the present data do not support the conclusion that DS shortens total procedure duration in this lesion subset.

Lower contrast use may be particularly relevant in patients with impaired renal function or diabetes, although the present study was not designed to assess contrast-associated acute kidney injury [11,24]. Likewise, the lower cumulative reference air kerma observed with DS should not be interpreted as evidence of shorter fluoroscopy time or reduced occupational radiation exposure, because neither fluoroscopy duration nor staff dosimetry was directly measured. Nevertheless, the lower cumulative reference air kerma remains a relevant procedural finding within the scope of the outcomes assessed in this study. Lower contrast use may also have implications for procedural resource utilization. However, no formal economic analysis was performed, and the present findings should not be interpreted as demonstrating cost-effectiveness.

The increasing feasibility of DS is closely linked to advances in stent technology. Contemporary DES platforms with thin struts, low crossing profiles, and high flexibility have improved stent deliverability and made DS increasingly feasible in suitable coronary anatomy [25]. Postdilatation was numerically, but not significantly, more frequent in the DS group. This finding should be interpreted cautiously. Postdilatation is a subsequent optimization step and does not alter the classification of the initial deployment strategy, but it may partly offset the reduction in procedural steps achieved by omitting predilatation.

### Limitations

This study has several limitations. Its retrospective, single-center, nonrandomized design introduces the possibility of selection bias and confounding by indication, because the choice between DS and PS was based on operator judgment and lesion suitability rather than random allocation. Although the adjusted analyses accounted for several measured clinical, lesion-related, procedural, and operator-related factors, residual and unmeasured confounding cannot be excluded. In particular, although lesion morphology was assessed for ACC/AHA classification, calcification burden, tortuosity, angulation, and bifurcation or ostial involvement were not recorded as separate graded variables, and quantitative stenosis severity and plaque burden were not available; these factors therefore could not be included as covariates. Systematic use of intracoronary imaging was likewise not consistently available for adjustment. Calendar year was not included in the adjusted models; temporal changes in equipment, practice patterns, or operator experience during the study period may therefore have influenced the findings, although all procedures were performed at a single center with contemporary drug-eluting stents by six experienced operators, and operator was included as a covariate. Different stent platforms were used during the study period, but the specific stent platform was not recorded in the analytic dataset and therefore could not be included in the adjusted analyses. This represents a potential source of residual confounding.

Lesion type was retrospectively classified by two investigators through review of stored angiograms and procedural reports, and neither independent blinded core-laboratory adjudication nor formal assessment of interobserver agreement was performed. Lesion length was based on visual angiographic estimation rather than quantitative coronary angiography. Although procedural records were reviewed for failed direct-stenting attempts and crossover, brief undocumented attempts at direct stent passage cannot be completely excluded owing to the retrospective design. A small number of patients contributed more than one PCI observation. This within-patient correlation was addressed using generalized estimating equations, and the findings were consistent in a sensitivity analysis restricted to one PCI observation per patient.

Radiation exposure was assessed using cumulative reference air kerma reported by the angiography system. Fluoroscopy time, kerma-area product, and occupational radiation exposure were not measured. Femoral access was used in the majority of procedures, which may limit the generalizability of the findings to centers with predominantly radial practice.

Clinical outcomes, including contrast-associated acute kidney injury, were not systematically analyzed, and the study was not designed or powered to establish equivalence or noninferiority in clinical safety or effectiveness between the two strategies. In addition, no formal health-economic analysis was performed; therefore, the present data do not permit any inference regarding cost-effectiveness.

## 5. Conclusions

In this retrospective observational study of elective PCI for Type A and B1 coronary lesions, direct stenting was associated with lower cumulative contrast volume and lower cumulative reference air kerma compared with predilatation followed by stenting. These associations remained significant after adjustment for measured clinical, lesion-related, procedural, and operator-related factors and were consistent in the sensitivity analysis restricted to one PCI observation per patient. Although median procedure duration was shorter with direct stenting in unadjusted comparisons, no statistically significant difference was observed after multivariable adjustment (adjusted difference −0.92 min, 95% CI −4.56 to 2.71; *p* = 0.619). Because treatment strategy was not randomly assigned and residual confounding cannot be excluded, these findings should be interpreted as observational associations rather than evidence of a causal benefit. The study was neither designed nor powered to establish equivalence or noninferiority in clinical safety or effectiveness between the two strategies. No conclusions regarding cost-effectiveness can be drawn, as no formal health-economic analysis was performed. Prospective, ideally randomized, studies using contemporary drug-eluting stents are needed to confirm these findings.

## Figures and Tables

**Table 1:** Baseline demographic, clinical, and laboratory characteristics at the PCI-observation level.

Variables	Group 1 (PS): n = 239 Observations	Group 2 (DS): n = 95 Observations	*p*-Value
Age (years)	59 (53–67)	61 (56–68)	0.070
Sex (F/M, %)	35.1%/64.9%	25.3%/74.7%	0.081
BMI (kg/m^2^)	28.6 (25.8–31.6)	28.4 (26.1–31.2)	0.797
Heart rate (beats/min)	69 (62–74)	69 (66–71)	0.815
SBP (mmHg)	133 (123–142)	135 (127–144)	0.090
DBP (mmHg)	75 (70–84)	78 (71–86)	0.151
Hypertension (%)	68.2%	69.5%	0.821
Diabetes mellitus (%)	30.1%	27.4%	0.618
Hyperlipidemia (%)	74.5%	71.6%	0.588
Smoking (%)	36.4%	36.8%	0.940
Family history of CVD (%)	23.4%	28.4%	0.341
COPD (%)	5.9%	6.3%	0.874
Atrial fibrillation (%)	2.1%	4.2%	0.281
Peripheral arterial disease (%)	3.8%	5.3%	0.538
History of CABG (%)	2.9%	2.1%	0.675
Heart failure (%)	4.2%	2.1%	0.608
Stroke (%)	3.3%	1.1%	0.243
Glucose (mg/dL)	110 (93–147)	121 (102–154)	0.106
Creatinine (mg/dL)	0.89 (0.74–1.02)	0.90 (0.78–1.07)	0.139
eGFR (mL/min/1.73 m^2^)	90 (74–100)	93 (90–98)	0.411
LDL-C (mg/dL)	95 (73–125)	101 (73–127)	0.827
HDL-C (mg/dL)	40 (34–47)	36 (31–45)	0.150
Triglycerides (mg/dL)	147 (106–235)	169 (112–235)	0.331
Hemoglobin (g/dL)	13.7 (12.2–14.7)	13.8 (12.7–14.8)	0.731
Leukocyte count (10^3^/μL)	7.95 (6.91–9.56)	8.16 (6.93–9.76)	0.362
Platelet count (10^3^/μL)	252 (214–286)	255 (206–284)	0.427
LVEF (%)	60 (60–62)	60 (55–65)	0.844

Data are presented at the PCI-observation level (n = 334 PCI observations from 324 patients); clinical and laboratory variables were recorded at the time of each procedure. Fasting glucose and lipid parameters were not available for all PCI observations; these variables were summarized using available data (available-case analysis). Variables included in the adjusted models and the procedural outcomes had no missing values. Continuous variables are presented as median (interquartile range) and were compared using the Mann–Whitney U test; categorical variables are presented as percentages and were compared using the chi-square or Fisher’s exact test, as appropriate. *p* values are unadjusted and do not account for within-patient clustering. **Abbreviations:** PS: Predilatation followed by Stenting group; DS: Direct Stenting group; F/M: Female/Male; BMI: Body Mass Index; SBP: Systolic Blood Pressure; DBP: Diastolic Blood Pressure; COPD: Chronic Obstructive Pulmonary Disease; CABG: Coronary Artery Bypass Grafting; CVD: cardiovascular disease; eGFR: Estimated Glomerular Filtration Rate; LDL-C: Low-Density Lipoprotein Cholesterol; HDL-C: High-Density Lipoprotein Cholesterol; LVEF: Left Ventricular Ejection Fraction.

**Table 2:** Angiographic and procedural characteristics at the PCI-observation level.

Variables	Group 1 (PS): n = 239 PCI Observations	Group 2 (DS): n = 95 PCI Observations	*p*-Value
Access site (F/R) (n)	219/20	90/5	0.331
Operator (1/2/3/4/5/6) (n)	33/49/56/33/34/34	20/10/21/13/20/11	0.133
Lesion type (A/B1) (n)	100/139	40/55	0.965
Treated vessel			0.221
-LAD (n)	98 (41.0%)	39 (41.1%)	
-LCX (n)	65 (27.2%)	28 (29.5%)	
-RCA (n)	58 (24.3%)	27 (28.4%)	
-IM (n)	6 (2.5%)	0 (0%)	
-Diagonal (n)	12 (5.0%)	1 (1.1%)	
Passive/support catheter (n)	14 (5.9%)	7 (7.4%)	0.608
Extra support wire (n)	12 (5.0%)	6 (6.3%)	0.636
Buddy wire (n)	9 (3.8%)	4 (4.2%)	0.850
Lesion length (mm)	12 (8–17)	12 (8–16)	0.606
Stent length (mm)	19 (16–23)	18 (16–20)	0.162
Stent diameter (mm)	3.00 (2.75–3.00)	3.00 (2.75–3.00)	0.234
Postdilatation (%)	30.5%	41.1%	0.066
Repeat postdilatation (%)	5.0%	7.4%	0.403
Procedure duration (min)	33 (27–39)	27 (26–36)	0.032
Contrast volume (mL)	150 (100–175)	125 (100–150)	0.009
Cumulative reference air kerma (mGy)	625 (478–896)	420 (262.5–613.5)	<0.001

Data are presented at the PCI-observation level (n = 334 PCI observations from 324 patients). Nine patients contributed repeated PCI observations (eight patients with two and one patient with three procedures). Continuous variables are presented as median (interquartile range) and were compared using the Mann–Whitney U test; categorical variables were compared using the chi-square or Fisher’s exact test, as appropriate. *p* values are unadjusted and do not account for within-patient clustering; adjusted analyses of the principal procedural outcomes are presented in Table 3. **Abbreviations:** PS: Predilatation followed by Stenting group; DS: Direct Stenting group; F/R: Femoral/Radial; LAD: Left Anterior Descending artery; LCX: Left Circumflex artery; RCA: Right Coronary Artery; IM: Intermediate artery.

**Table 3:** Adjusted differences in the principal procedural outcomes between direct stenting and predilatation followed by stenting.

Outcome	Principal GEE Analysis, Adjusted Difference (95% CI)	*p*-Value	Sensitivity Analysis *, Adjusted Difference (95% CI)	*p*-Value
Procedure duration (min)	−0.92 (−4.56 to 2.71)	0.619	−1.00 (−4.44 to 2.44)	0.569
Cumulative contrast volume (mL)	−18.12 (−28.08 to −8.17)	<0.001	−16.05 (−25.99 to −6.10)	0.002
Cumulative reference air kerma (mGy)	−223.54 (−259.98 to −187.11)	<0.001	−220.42 (−256.46 to −184.37)	<0.001

Differences are expressed as direct stenting minus predilatation followed by stenting; negative values indicate lower values with direct stenting. Principal analysis: generalized estimating equations (n = 334 PCI observations; identity link, exchangeable working correlation structure, robust standard errors, clustered by patient). Sensitivity analysis: multivariable linear regression restricted to the first recorded PCI observation of each patient (n = 324), with heteroscedasticity-robust standard errors. Both models were adjusted for age, sex, diabetes mellitus, estimated glomerular filtration rate, lesion type, lesion length, treated vessel, vascular access, and operator. Holm-adjusted *p* values for the principal analysis were 0.619, <0.001, and <0.001, respectively. * Sensitivity analysis was restricted to the first PCI observation per patient (n = 324).

## Data Availability

The data associated with this study are available from the corresponding author upon reasonable request.

## References

[1] Parker W.A.E. (2025). Evolution of coronary stents: Innovations, antithrombotic strategies and future directions. Heart.

[2] Lee D.H., de la Torre Hernandez J.M. (2018). The Newest Generation of Drug-eluting Stents and Beyond. Eur. Cardiol..

[3] Saito Y., Kobayashi Y. (2026). Advances in Technology and Technique in Percutaneous Coronary Intervention: A Clinical Review. Intern Med..

[4] Martínez-Elbal L., Ruiz-Nodar J.M., Zueco J., López-Minguez J.R., Moreu J., Calvo I., Ramirez J.A., Alonso M., Vazquez N., Lezaun R. (2002). Direct coronary stenting versus stenting with balloon pre-dilation: Immediate and follow-up results of a multicentre, prospective, randomized study. The DISCO trial. DIrect Stenting of COronary Arteries. Eur. Heart J..

[5] Alidoosti M., Salarifar M., Kassaian S.E., Zeinali A.M., Fathollahi M.S., Dehkordi M.R. (2008). In-hospital and mid-term adverse clinical outcomes of a direct stenting strategy versus stenting after pre-dilatation for the treatment of coronary artery lesions. Cardiovasc J. Afr..

[6] Ökmen E., Çam N. (2002). Direct stent implantation: Feasibility, advantages and disadvantages. Anatol. J. Cardiol..

[7] Wu X., Liu Z., Huang H., Wu M., Huang H., Wang L. (2025). Intravascular ultrasound assessment of stent edge restenosis mechanisms and treatment outcomes following percutaneous coronary intervention. Sci. Rep..

[8] Shahzad K., Mallick N.H., Amin S., Ahmad S., Ullah H. (2020). Direct Stenting Versus Balloon Pre-Dilatation In Elective Percutaneous Coronary Intervention. J. Ayub Med. Coll. Abbottabad.

[9] Aldujeli A., Haq A., Tsai T.Y., Grabauskyte I., Tatarunas V., Briedis K., Rana S., Unikas R., Hamadeh A., Serruys P.W. (2023). The impact of primary percutaneous coronary intervention strategies during ST-elevation myocardial infarction on the prevalence of coronary microvascular dysfunction. Sci. Rep..

[10] Vogel R.F., Delewi R., Wilschut J.M., Lemmert M.E., Diletti R., van Vliet R., van der Waarden N.W.P.L., Nuis R.-J., Paradies V., Alexopoulos D. (2023). Direct Stenting versus Conventional Stenting in Patients with ST-Segment Elevation Myocardial Infarction-A COMPARE CRUSH Sub-Study. J. Clin. Med..

[11] Hennessey B., Shabbir A., Travieso A., Gonzalo N., Escaned J. (2023). Procedural and Technological Innovations Facilitating Ultra-low Contrast Percutaneous Coronary Interventions. Interv. Cardiol..

[12] Gutiérrez-Barrios A., Camacho-Galán H., Medina-Camacho F., Cañadas-Pruaño D., Jimenez-Moreno A., Calle-Perez G., Vázquez-García R. (2019). Effective Reduction of Radiation Exposure during Cardiac Catheterization. Tex. Heart Inst. J..

[13] Barbato E., Gallinoro E., Abdel-Wahab M., Andreini D., Carrié D., Di Mario C., Dudek D., Escaned J., Fajadet J., Guagliumi G. (2023). Management strategies for heavily calcified coronary stenoses: An EAPCI clinical consensus statement in collaboration with the EURO4C-PCR group. Eur. Heart J..

[14] Kahe F., Sharfaei S., Pitliya A., Jafarizade M., Seifirad S., Habibi S., Chi G. (2020). Coronary artery tortuosity: A narrative review. Coron. Artery Dis..

[15] Leone A., Simonetti F., Avvedimento M., Angellotti D., Immobile Molaro M., Franzone A., Esposito G., Piccolo R. (2022). Ultrathin Struts Drug-Eluting Stents: A State-of-the-Art Review. J. Pers. Med..

[16] Magalhaes M.A., Minha S., Lhermusier T., Pendyala L., Escarcega R.O., Baker N.C., Torguson R., Satler L.F., Pichard A., Waksman R. (2017). Does direct stenting with drug-eluting stents improve outcome? A meta-analysis of 10,900 patients. Catheter Cardiovasc Interv..

[17] Abbott J.D., Earl T., Vlachos H.E., Selzer F., Vaidya K.A., Romero A., Kip K.E., Williams D.O. (2012). Direct stenting compared to balloon predilation in drug-eluting stents: One-year outcomes from the DEScover Registry. Catheter Cardiovasc Interv..

[18] Remkes W.S., Somi S., Roolvink V., Rasoul S., Ottervanger J.P., Gosselink A.T.M., Hoorntje J.C.A., Dambrink J.-H.E., de Boer M.-J., Suryapranata H. (2014). Direct drug-eluting stenting to reduce stent restenosis: A randomized comparison of direct stent implantation to conventional stenting with pre-dilation or provisional stenting in elective PCI patients. JACC Cardiovasc Interv..

[19] Konigstein M., Redfors B., Zhang Z., Kotinkaduwa L.N., Mintz G.S., Smits P.C., Serruys P.W., von Birgelen C., Madhavan M.V., Golomb M. (2022). Utility of the ACC/AHA Lesion Classification to Predict Outcomes After Contemporary DES Treatment: Individual Patient Data Pooled Analysis From 7 Randomized Trials. J. Am. Heart Assoc..

[20] Secco G.G., Ghione M., Mattesini A., Dall’Ara G., Ghilencea L., Kilickesmez K., De Luca G., Fattori R., Parisi R., Marino P.N. (2016). Very high-pressure dilatation for undilatable coronary lesions: Indications and results with a new dedicated balloon. EuroIntervention.

[21] Lee M.S., Park S.J., Kandzari D.E., Kirtane A.J., Fearon W.F., Brilakis E.S., Vermeersch P., Kim Y.-H., Waksman R., Mehilli J. (2011). Saphenous vein graft intervention. JACC Cardiovasc Interv..

[22] Caluk J., Osmanovic E., Barakovic F., Kusljugic Z., Terzic I., Caluk S., Sofic A. (2010). Direct coronary stenting in reducing radiation and radiocontrast consumption. Radiol. Oncol..

[23] Kovar L.I., Monrad E.S., Sherman W., Kunchithapatham S., Ravi K.L., Gotsis W., Silverman G., Brown D.L. (2001). A randomized trial of stenting with or without balloon predilation for the treatment of coronary artery disease. Am. Heart J..

[24] Virani S.S., Newby L.K., Arnold S.V., Bittner V., Brewer L.C., Demeter S.H., Dixon D.L., Fearon W.F., Hess B., Johnson H.M. (2023). 2023 AHA/ACC/ACCP/ASPC/NLA/PCNA Guideline for the Management of Patients with Chronic Coronary Disease: A Report of the American Heart Association/American College of Cardiology Joint Committee on Clinical Practice Guidelines. Circulation.

[25] Noad R.L., Hanratty C.G., Walsh S.J. (2014). Clinical Impact of Stent Design. Interv. Cardiol..

